# Changes in Lipoprotein Particles in the Blood Serum of Patients with Lichen Planus

**DOI:** 10.3390/metabo13010091

**Published:** 2023-01-06

**Authors:** Liis Ilves, Aigar Ottas, Liisi Raam, Mihkel Zilmer, Tanel Traks, Viljar Jaks, Külli Kingo

**Affiliations:** 1 Department of Dermatology and Venereology, University of Tartu, 50417 Tartu, Estonia; 2 Dermatology Clinic, Tartu University Hospital, 50417 Tartu, Estonia; 3 Department of Biochemistry, Institute of Biomedicine and Translational Medicine, University of Tartu, 50411 Tartu, Estonia; 4 Centre of Excellence for Genomics and Translational Medicine, University of Tartu, 50411 Tartu, Estonia; 5 Faculty of Medicine, Institute of Clinical Medicine, University of Tartu, 50411 Tartu, Estonia; 6 Department of Cell Biology, Institute of Molecular and Cell Biology, University of Tartu, 51010 Tartu, Estonia

**Keywords:** lichen planus, dermatology, lipids

## Abstract

Lichen planus is a chronic inflammatory mucocutaneous disease that belongs to the group of papulosquamous skin diseases among diseases like psoriasis, a widely studied disease in dermatology. The aim of the study was to identify the changes between the blood sera of lichen planus patients and healthy controls to widen the knowledge about the metabolomic aspect of lichen planus and gain a better understanding about the pathophysiology of the disease. We used high-throughput nuclear magnetic resonance (NMR) spectroscopy to measure the levels of blood serum metabolites, lipoproteins and lipoprotein particles. Dyslipidemia has relatively recently been shown to be one of the comorbidities of lichen planus, but the changes in the components of lipoproteins have not been described yet. We found statistically significant changes in the concentrations of 16 markers regarding lipoproteins, which included the components of intermediate-density lipoproteins, low-density lipoproteins and large low-density lipoproteins. We propose that the detected changes may increase the risk for specific comorbidities (e.g., dyslipidemia) and resulting cardiovascular diseases, as the turnover and hepatic uptake of the altered/modified lipoprotein particles are disturbed.

## 1. Introduction

Lichen planus (LP) is a chronic inflammatory mucocutaneous disease that belongs to the group of papulosquamous skin diseases together with psoriasis and pityriasis rubra pilaris among others [1]. In addition to skin and mucous membranes, hair and nails might be affected [2]. A distinctive feature of this disease is the presence of extremely pruritic flat polygonal violaceous papules with characteristic Wickham striae. The rash is typically located on the flexor surfaces on the wrists, shins and sacral area, but can also be disseminated and involve the whole body [3]. LP has a substantial negative impact on the quality of life of LP patients. The patients with genital, ungual and cutaneous LP are most severely affected, and LP patients have lower self-esteem [4,5]. Jalenques et al. have shown that the signs of depression and anxiety are highly prevalent in LP patients (27% and 28%, respectively) [6].

External factors (e.g., stress, trauma, infections) and genetics play an important role in the development and persistence of LP. There is a genetic predisposition, especially in patients with specific HLA haplotypes like HLA-Bw57, HLA-B27 and HLA-DR [7,8,9]. The pathophysiology of the disease involves the migration of CD8^+^ T-lymphocytes to the dermoepidermal junction and the induction of apoptosis in basal keratinocytes. The cytokines that take part in the development of the disease are interferon γ (IFN-γ), tumor necrosis factor α (TNF-α), interleukin 6 (IL-6) and IL-8. Additionally, an increase in local angiogenesis has been found [2,10,11]. 

The most common comorbidities of LP are hepatitis C virus infection (HCV) and thyroid disease [2,12]. Recently, metabolic syndrome (MS), type 2 diabetes (T2D) and dyslipidemia have also been reported to be associated with LP [13,14,15,16,17,18,19]. Additionally, LP patients have increased risk for cardiovascular diseases [20,21]. 

Metabolomic studies have been used to increase our understanding of various diseases, including inflammatory skin diseases like psoriasis and atopic dermatitis [22,23]. To our best knowledge, the data regarding the metabolomics of skin or blood serum of LP patients with skin involvement are lacking. However, there exist a handful of studies addressing the metabolomics of oral LP (OLP). In the case of erosive OLP, which is an oral potentially malignant disorder, the alterations in serum metabolite levels indicated the increased presence of oxidative stress, apoptosis, neutrophil dysfunction and inflammation [24,25]. In the mucosal biopsies of OLP, the altered ratio of linoleic acid-derived oxylipins indicated the activation of cyclooxygenase-2 (COX-2) and related cyclooxygenases, which were suggested to contribute to the symptomatology of LP, especially to the pain associated with the disease [26]. 

The alterations of low-density lipoprotein cholesterol (LDL-C) and other forms of lipoproteins have been described in LP patients’ blood (e.g., an increase in LDL-C and a decrease in high-density lipoprotein cholesterol (HDL-C)) [15], but information regarding the sizes of subfractions of blood lipoproteins has not been published yet. Consequently, information about lipoprotein subfractions in LP may have a value for widening the knowledge about the pathophysiology and cardiovascular risk of the disease. We hypothesize that the changes in blood serum lipoproteins and metabolites contribute to the manifestation of LP and its comorbidities.

In order to expand the knowledge about the metabolomic aspect and cardiovascular impact of LP and gain better understanding about the pathophysiology of this disease, we measured the blood serum lipoproteins and metabolites using high-throughput NMR spectroscopy. The usage of high-throughput NMR spectroscopy for measuring blood lipoproteins has good correlation with traditional/routine clinical chemical measurements of blood lipoproteins levels (personal communication from Nightingale Health OYJ, where the measurement was done) and additionally gives more detailed measurements of lipoprotein particle sizes. This allows us to clarify if the changes in the particles have a role in the development of the disease.

## 2. Materials and Methods

### 2.1. Volunteer Recruitment

Patients with cutaneous LP who had classic skin involvement were recruited from the Tartu University Hospital at the Clinic of Dermatology in 2021 (10 men, 22 women, 19–80 years old, median age 56.5 years). Healthy age- and sex-matched controls (HC) were recruited from the Tartu University Hospital at the Clinic of Dermatology (10 men, 22 women, 21–80 years old, median age 56 years). Diagnosed metabolic and cardiovascular comorbidities of the cohorts are presented in Table S1. The patients and the controls were Caucasians of Eastern European descent.

### 2.2. Blood Samples

Blood sera were collected before the first meal of the day using 5 mL Vacutainer (REF 367614) tubes that contained micronized silica particles to accelerate the clotting process. The collected blood samples were left to clot at room temperature for one hour, subsequently centrifuged at 1300× *g* for 20 min. The serum was pipetted into 300 μL aliquots and was stored at −80 °C until measurement.

### 2.3. Mass-Spectrometric Analysis

The serum samples were shipped on dry ice to Nightingale Health OYJ where they were measured using high-throughput NMR spectroscopy. The measurement enables the concurrent quantification of 248 metabolites and particles, which include various lipids, lipoproteins, fatty acids and multiple low-molecular-weight metabolites (e.g., amino acids, ketone bodies and glycolysis intermediates). The concentrations are quantified in molar units. More exact details about the measurement are explained in a review article [27]. 

### 2.4. Data Analysis

The data was analyzed using R version 4.1.3. [28]. The dataset was scaled and outliers that had SD > 4 from the mean metabolite and particle concentrations were removed. Wilcoxon–Mann–Whitney test was used to determine the metabolites and particles that differed significantly between LP and HC. The principal component analysis showed a total of 19 principal components (PCs) explaining >95% of variability in the data. The multiple-testing threshold was set according to *p* < 0.05/19 PCs; i.e., *p* < 0.0029.

## 3. Results

In our study, 16 markers regarding lipoproteins differed statistically significantly between the blood sera of LP patients and healthy controls (HC). We found an increase in the levels of components of intermediate-density lipoprotein (IDL), low-density lipoprotein (LDL) and large LDL (L-LDL) in the blood sera of LP patients when compared to the HC. It should be noted that lipoproteins of different classes are divided into subclasses depending on their size, thus similar components are present in IDL, LDL and L-LDL lipoproteins. 

**Figure 1:**
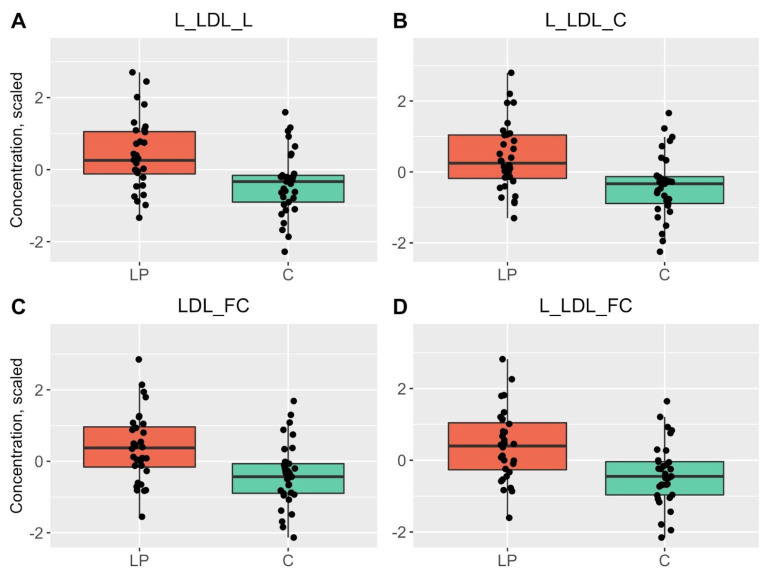
Boxplots of the top four statistically significant results that had differences between the blood samples of LP patients (red) and HC (green). *p* < 0.0029 is considered statistically significant.

In IDL, the concentrations of phospholipids (PL), cholesteryl esters (CE), total cholesterol (TC) and free cholesterol (FC) were changed. The concentrations of total lipids, TC, FC, CE and PL were elevated in L-LDL and FC, TC and total lipids in LDL. Additionally, clinical value of LDL cholesterol (calculated as LDL-C + IDL-C + cholesterol in very small very-low-density lipoproteins × 0.15) and the levels of TC, total FC and total CE were also increased in LP patients (Table 1, Figure 1 and Figures S1–S3).

Additionally, smaller changes in the composition of high-density lipoproteins (HDL) and VLDL as well as in the concentration of non-HDL cholesterol, sphingomyelins, linoleic acid (LA), polyunsaturated fatty acids (PUFA), omega-6 fatty acids and amino acid leucine (Leu) were also detected; however, the changes were not statistically significant (Table S2).

In addition, we analysed ratios that relate to cardiovascular diseases and T2D, which are known comorbidities of LP. However, the ratios of HDL-C to ApoA1, LDL-C to HDL-C, total triglycerides (TG) to HDL-C, total FC to HDL-C, non-HDL-C to HDL-C and LDL-C to apolipoprotein B (ApoB) were not altered in LP patients when compared to the HC. 

We also studied other metabolites like glycolysis related metabolites, ketone bodies and other apolipoproteins and amino acids, but found no changes between the levels in LP and HC patients.

## 4. Discussion

Significant association between lichen planus (LP) and metabolic syndrome (MS) has recently been established, especially in the severe form of LP [15,29]. MS, as well as one of its components—dyslipidemia—have both been previously shown to be comorbidities of LP, and vice versa, chronic inflammatory skin diseases increase the risk for MS and dyslipidemia. Dyslipidemias are a group of metabolic derangements characterized by various deviations in lipid levels (e.g., increased LDL-C, cholesterol and TG levels and decreased HDL-C levels) [5,30,31]. In the present study, we found increased levels of cholesterol (C), cholesteryl esters (CE), free cholesterol (FC), total lipids (L) and phospholipids (PL) in L-LDL, LDL and IDL particles in the blood sera of LP patients. In our study group, the prevalence of previously laboratory-confirmed dyslipidemia occurred less frequently (Table S1) than we would have expected based on the results of analysing the data and previously published studies, which means LP patients should routinely be screened for dyslipidemia. 

The classical structure of lipoproteins is well-known. Lipoproteins are divided into subclasses based on the density and composition. All lipoproteins as particles consist mainly of apoproteins, TG-s, phospholipids, CE-s and FC-s. The size and density of lipoprotein subclasses depend on the amounts of abovementioned components. The particle subclasses can be divided into several subfractions (e.g., large, small) mainly based on different ratios, but also different amounts of previously mentioned components. The central apoprotein in VLDL and LDL is ApoB-100, and ApoA in HDL [32,33]. Apolipoproteins serve as ligands for lipoprotein receptors and cofactors for enzymes in lipid metabolism; they maintain the structure of the lipoprotein particles and guide the formation of lipoproteins, but they also have more specific functions (e.g., ApoA-I and endogenous ApoE prevent inflammation and oxidative stress) [33,34]. 

There exist data regarding the changes in lipid and lipoprotein levels in the blood of the patients with LP, but the specific alterations in the structure of lipoproteins were not known. To our knowledge, we demonstrated for the first time that there exist alterations in the composition of lipoproteins isolated from the blood sera of LP patients. It has previously been found that the patients with LP have increased concentrations of total cholesterol (TC), LDL-C and TG and decreased levels of HDL-C. In addition, LP patients have also higher TC/HDL-C and LDL-C/HDL-C ratios, concomitantly an increase in C-reactive protein (CRP) as well as malondialdehyde levels and decrease in catalase activity were detected, which pointed to the increase in inflammation processes and presence of oxidative stress [15]. There was also a significant decrease in HDL-C (*p* = 0.003) and increase in Castelli’s atherogenic index (total cholesterol/HDL cholesterol; *p* = 0.005) in OLP patients [35]. 

As IDL, L-LDL and LDL are all connected to lipid transport and are pro-atherogenic (which also depends on the size of the particles, e.g., smaller and denser particles are more easily able to penetrate into subendothelial space), changes in their composition may alter the appearance and course of comorbidities. Grammer et al. showed the increased risk for all-cause mortality, including cardiovascular, in patients with higher L-LDL and small-LDL (S-LDL) levels compared to intermediate-size LDL levels. Additionally, in patients with elevated L-LDL levels, they also found increased IL-6 and CRP levels [36]. As mentioned above, both of them are also found to be elevated in LP patients [11,15,18]. 

Lipoproteins are physiologically heterogenic and their structure is altered in pathologic conditions. In our study, the changes in the structure of LDL, L-LDL and IDL were statistically significant between LP patients and HC. The composition of LDL particles also affects the development of atherosclerotic cardiovascular disease. Ruuth et al. showed that more sphingolipids and less phosphatidylcholines in LDL particles increase the aggregation of LDL, which was associated with future cardiovascular death [37]. We hypothesize that the changes detected in this work act in concert to affect the function of lipoproteins, which in turn increases the risk for cardiovascular events. 

We found small, however, statistically insignificant changes in the composition of HDL. Quantitative and qualitative molecular changes of HDL have been described in diseases like diabetes mellitus, coronary artery disease, chronic kidney disease and rheumatic diseases [38,39]. Normally, HDL protects against cardiovascular disease as it removes excess cholesterol from macrophages; has antioxidative, anti-thrombotic, anti-apoptotic and anti-inflammatory properties; modulates endothelial function and stimulates the movement of endothelial cells. In type 1 diabetes (T1D), HDL levels may be normal, but the risk for cardiovascular events has risen, nevertheless. It has been suggested that changes in functional properties of HDL may contribute to the loss of its protective properties [33,40,41].

Gardner et al. have characterized the changes in composition in HDL in patients with T2D and coronary heart disease (CHD) and the changes in its function [38,42]. In T2D patients, they found an increase in TG-rich particles and a decrease in large and very large particles. In both T2D patients and CHD patients, the concentrations in lipid species were altered when compared to the healthy controls; in T2D, the levels of 71 lipid species were decreased and 14 were increased compared to the controls, and in CHD patients, the concentrations of 5 lipid species were decreased and 4 increased. Functionally, HDL in T2D patients was described as having lower anti-apoptotic activity against human aortic endothelial cells [42]. Although the changes in the composition of HDL were not statistically significant in our study, the abovementioned findings indicate that changes in the composition of lipoprotein particles lead to alterations in their function. 

In conclusion, we found that the composition of lipoproteins was altered in the blood serum of patients with LP. The composition of lipoproteins has been shown to alter their function, and we propose that the detected changes may increase the risk of LP patients for specific comorbidities like dyslipidemia, MS and T2D, and vice versa. As the turnover and hepatic uptake of the altered/modified LDL and IDL particles is disturbed, resulting increased cardiovascular risk can be considered. Further studies to clarify the relationship between the composition and function of lipoproteins are needed in order to unravel their role in the development of LP and its comorbidities. However, the results of the present study support the need for screening for dyslipidemia in patients with LP.

## Figures and Tables

**Table 1:** Statistically significant differences in lipoprotein particles and metabolites between blood serum samples obtained from patients suffering from LP and HC.

Composition of Lipoprotein Particles and Metabolites	Mean for LP	Mean for HC	Wilcox-Test LP vs. HC, *p* < 0.0029
Large LDL			
L-LDL-L	0.4	−0.39	0.0007
L-LDL-C	0.41	−0.4	0.0008
L-LDL-FC	0.41	−0.39	0.0008
L-LDL-CE	0.4	−0.39	0.001
L-LDL-PL	0.4	−0.38	0.0011
LDL			
LDL-FC	0.39	−0.38	0.0008
LDL-C	0.38	−0.37	0.0024
LDL-L	0.38	−0.37	0.0027
IDL			
IDL-PL	0.4	−0.39	0.0013
IDL-CE	0.4	−0.39	0.0021
IDL-C	0.4	−0.39	0.0023
IDL-FC	0.39	−0.38	0.0027
Clinical LDL cholesterol and its values			
Clinical LDL-C *	0.39	−0.38	0.0014
Total C	0.41	−0.4	0.0016
Cholesteryl esters			
Total CE	0.41	−0.39	0.0013
Free cholesterol			
Total FC	0.4	−0.39	0.0028

C–cholesterol; CE–cholesteryl esters; FC–free cholesterol; HC–healthy controls; IDL–intermediate-density lipoprotein; L-–large; -L–total lipids; LDL–low-density lipoprotein; LP–lichen planus; PL–phospholipids. * Clinical LDL-C can be obtained from the NMR-based measures with the equation clinical LDL-C = LDL-C + IDL-C + XS-VLDL × 0.15.

## Data Availability

All data generated or analyzed during this study are included in this published article.

## Supplementary Materials

The following supporting information can be downloaded at: https://www.mdpi.com/article/10.3390/metabo13010091/s1, Table S1: Diagnosed metabolic and cardiovascular comorbidities of LP patients and HC; Table S2: Metabolite and lipoprotein particle concentrations that did not differ statistically significantly between blood serum samples obtained from patients suffering from LP, and HC, but had a trend toward alterations (*p* < 0.05); Figure S1: Boxplots of the metabolites and lipoprotein particles that had statistically significant differences between the blood samples of LP patients and HC; Figure S2: Boxplots of the metabolites and lipoprotein particles that had statistically significant differences between the blood samples of LP patients and HC; Figure S3: Boxplots of the metabolites and lipoprotein particles that had statistically significant differences between the blood samples of LP patients and HC.
