# Supplementary material for: Changes in Lipoprotein Particles in the Blood Serum of Patients with Lichen Planus
Source: Metabolites. 2023 Jan 6;13(1):91. doi: 10.3390/metabo13010091 (PMC9866152; doi:10.3390/metabo13010091)
Supplement: Supplementary file 1 [file metabolites-13-00091-s001.zip › metabolites-2113632-supplementary.pdf]

Table S1. Diagnosed metabolic and cardiovascular comorbidities of LP patients and HC.

Diagnosis	Number of LP patients (%)	Number of HC (%)
**Cardiovascular**		
Hypertension	16 (50)	13 (40.6)
Atherosclerosis	2 (6.3)	1 (3.1)
Stenocardia	1 (3.1)	
Congestive heart failure	1 (3.1)	1 (3.1)
Atrial fibrillation	1 (3.1)	
**Metabolic**		
Type 2 diabetes	2 (6.3)	3 (9.4)
Hypothyroidism	4 (12.5)	
Pure hypercholesterolemia		1 (3.1)
**Infectious**		
Chronic hepatitis C infection	2 (6.3)	

Table S2. Metabolite and lipoprotein particle concentrations that did not differ statistically significantly between blood serum samples obtained from patients suffering from LP, and HC, but had a trend toward alterations ($p<0.05$).

Metabolites and lipoprotein particles	Mean for LP	Mean for HC	Wilcox-test LP vs. HC, $p < 0.05$
**Very small VLDL**			
XS-VLDL-CE	0.38	-0.37	0.0048
XS-VLDL-C	0.36	-0.35	0.0086
XS-VLDL-P	0.19	-0.31	0.0309
XS-VLDL-L	0.19	-0.31	0.0388
XS-VLDL-FC	0.19	-0.31	0.0388
**Small VLDL**			
S-VLDL-FC	0.32	-0.31	0.0148
S-VLDL-PL	0.31	-0.3	0.0206
S-VLDL-C	0.3	-0.29	0.0313
S-VLDL-L	0.28	-0.27	0.0323
S-VLDL-P	0.26	-0.26	0.0369
S-VLDL-CE	0.28	-0.27	0.0478
**Medium VLDL**			
M-VLDL-FC	0.34	-0.33	0.0062
M-VLDL-CE	0.36	-0.35	0.0064
M-VLDL-C	0.36	-0.35	0.0076
M-VLDL-PL	0.32	-0.31	0.0127
M-VLDL-P	0.31	-0.3	0.0178

M-VLDL-L	0.3	-0.29	0.0191
**Large VLDL**			
L-VLDL-CE	0.27	-0.26	0.0382
L-VLDL-C	0.25	-0.24	0.0449
**Very large VLDL**			
XXL-VLDL-TG	0.11	-0.29	0.0302
XXL-VLDL-PL	0.11	-0.28	0.0346
XXL-VLDL-FC	0.12	-0.29	0.0369
XXL-VLDL-P	0.14	-0.3	0.0394
**VLDL**			
″XXL-VLDL-P" + "XL-VLDL-P" + "L-VLDL-P" + "M-VLDL-P" + ″S-VLDL-P" + "XS-VLDL-P"	0.3	-0.29	0.0246
**IDL**			
IDL-L	0.39	-0.38	0.0029
IDL-P	0.35	-0.34	0.01
**LDL**			
LDL-CE	0.38	-0.37	0.0029
LDL-P	0.35	-0.34	0.0093
LDL-PL	0.37	-0.36	0.0037
“L-LDL-P" + "M-LDL-P" + "S-LDL-P"	0.35	-0.34	0.0093
**Small LDL**			
S-LDL-FC	0.33	-0.32	0.0076
S-LDL-C	0.32	-0.31	0.0153
S-LDL-P	0.31	-0.3	0.0185
S-LDL-PL	0.29	-0.28	0.0185
S-LDL-CE	0.31	-0.3	0.0191
S-LDL-L	0.31	-0.3	0.0206
**Medium LDL**			
M-LDL-FC	0.37	-0.36	0.0029
M-LDL-L	0.34	-0.33	0.0109
M-LDL-C	0.33	-0.32	0.0109
M-LDL-PL	0.34	-0.33	0.0127
M-LDL-CE	0.32	-0.31	0.0165
M-LDL-P	0.26	-0.25	0.0394
**Large LDL**			
L-LDL-P	0.38	-0.37	0.0042

**Small HDL**			
S-HDL-FC	0.34	-0.33	0.0052
S-HDL-PL	0.28	-0.27	0.0097
S-HDL-L	0.3	-0.29	0.0153
S-HDL-P	0.3	-0.3	0.0178
S-HDL-C	0.28	-0.27	0.0246
**Medium HDL**			
M-HDL-TG	0.28	-0.28	0.0357
**HDL**			
"XL-HDL-P" + "L-HDL-P" + "M-HDL-P" + "S-HDL-P"	0.24	-0.23	0.0263
**Cholesterol**			
non-HDL-C	0.39	-0.38	0.0034
Remnant cholesterol	0.38	-0.37	0.0048
VLDL-C	0.32	-0.31	0.0159
**Total lipids**			
Total-L	0.39	-0.38	0.0035
VLDL-L	0.25	-0.25	0.0302
**Phospholipids**			
Total-PL	0.38	-0.36	0.0067
VLDL-PL	0.28	-0.27	0.0292
**Cholesteryl esters**			
VLDL-CE	0.33	-0.32	0.0117
**Free cholesterol**			
VLDL-FC	0.3	-0.29	0.0171
**Lipoprotein particle concentrations**			
Total concentration of lipoprotein particles	0.29	-0.28	0.0113
Concentration of VLDL particles	0.3	-0.29	0.0246
Concentration of HDL particles	0.24	-0.23	0.0263
**Other lipids**			
Cholines	0.35	-0.34	0.0064
Phosphoglycerides	0.35	-0.34	0.0057
Phosphatidylcholines	0.34	-0.33	0.0086
Sphingomyelins	0.29	-0.28	0.0206
**Apolipoproteins**			
Apolipoprotein B	0.35	-0.34	0.0073

**Fatty acids**			
Polyunsaturated fatty acids	0.35	-0.34	0.007
Saturated fatty acids	0.34	-0.33	0.0086
Total fatty acids	0.33	-0.32	0.0093
Omega-6 fatty acids	0.35	-0.33	0.01
Linoleic acid	0.32	-0.31	0.0229
Omega-3 fatty acids	0.12	-0.24	0.0261
**Branched-chain amino acids**			
Leucine	0.15	-0.14	0.0478
**Fluid balance**			
Albumin	0.33	-0.32	0.0037
**Relative lipoprotein lipid concentrations**			
Cholesterol to total lipids ratio in L-LDL	0.33	-0.32	0.0097
Cholesteryl esters to total lipids ratio in L-LDL	0.32	-0.31	0.0153
Phospholipids to total lipids ratio in L-LDL	-0.3	0.29	0.0221
Cholesterol to total lipids ratio in L-HDL	-0.24	0.23	0.0421

C – cholesterol; CE – cholesteryl esters; FC – free cholesterol; HC – healthy controls; IDL – intermediate-density lipoprotein; L- – large; -L – total lipids; LDL – low-density lipoprotein; LP – lichen planus; M- – medium; -P – particles; PL – phospholipids; S- – small; XL- – very large; XS- – very small; XXL- – extremely large.

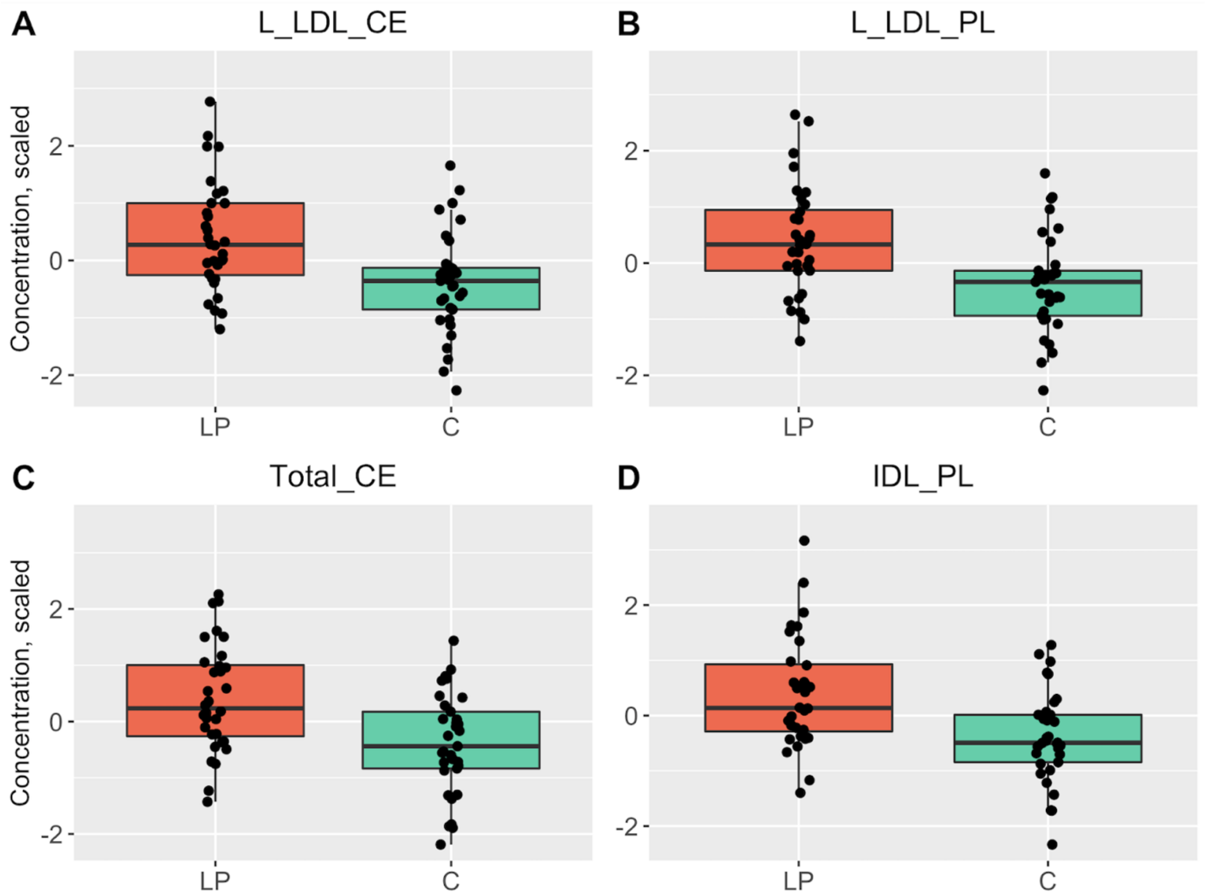

Figure S1. Boxplots of the metabolites and lipoprotein particles that had statistically significant differences between the blood samples of LP patients and HC.

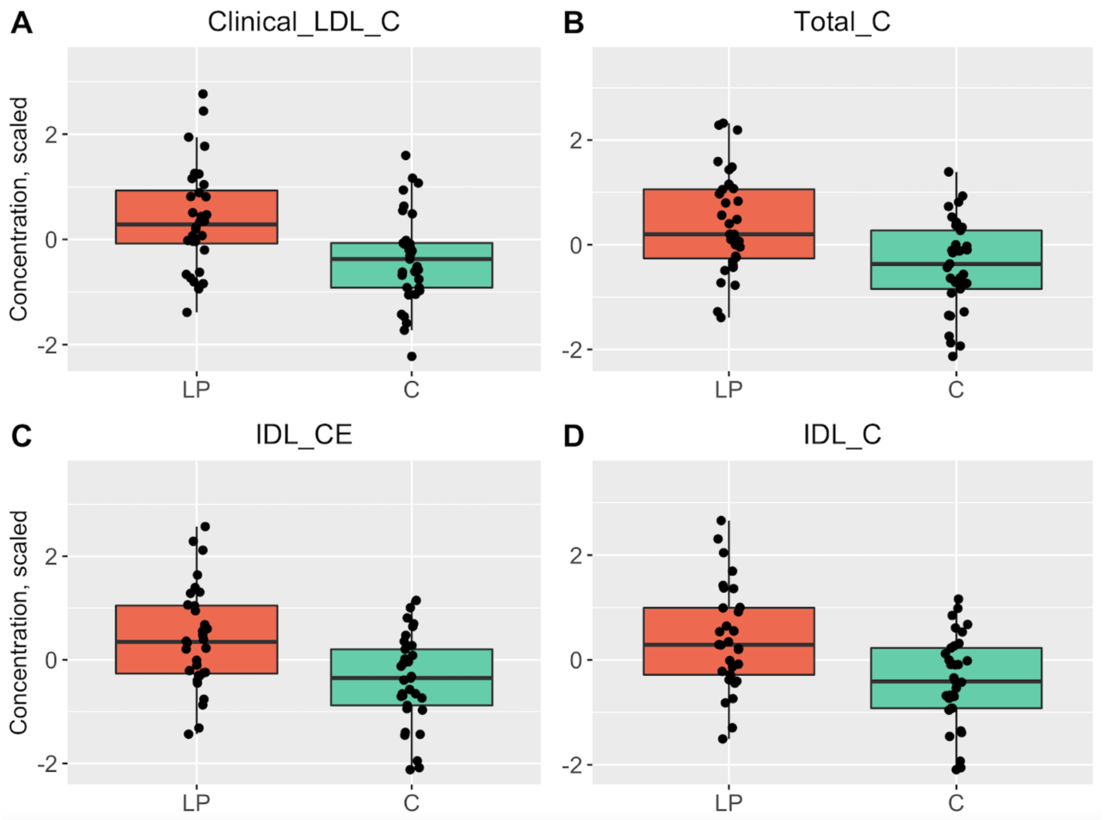

Figure S2. Boxplots of the metabolites and lipoprotein particles that had statistically significant differences between the blood samples of LP patients and HC.

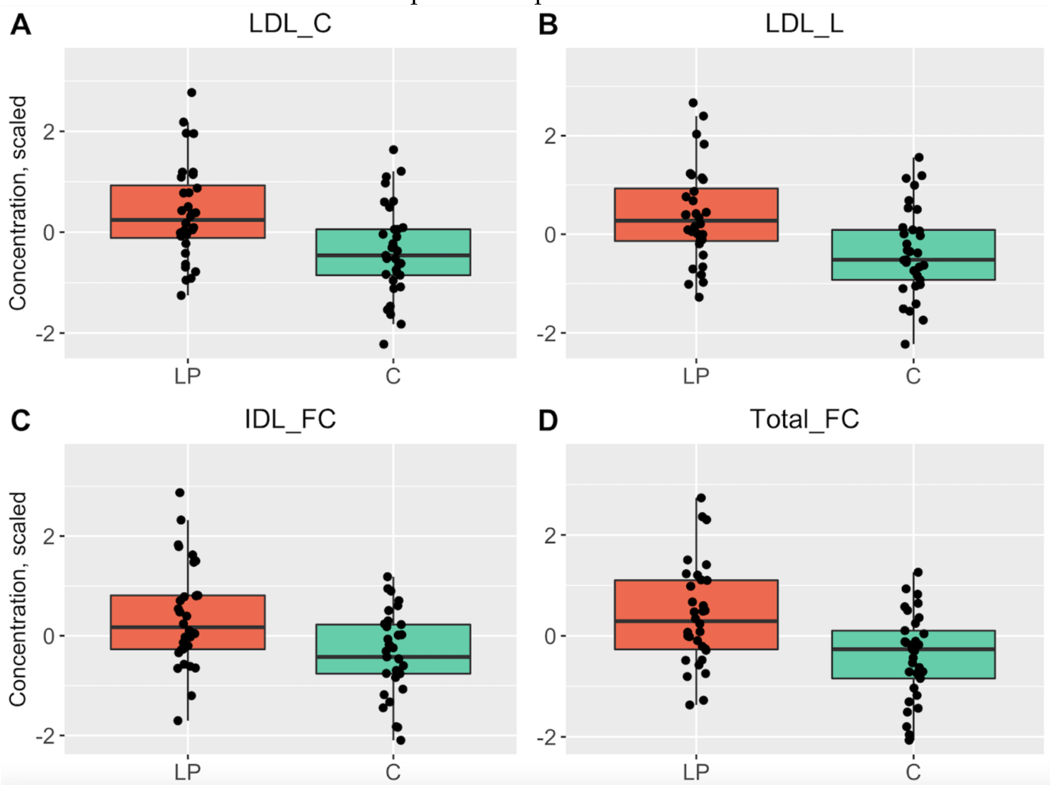

Figure S3. Boxplots of the metabolites and lipoprotein particles that had statistically significant differences between the blood samples of LP patients and HC.
